# Supplementary material for: Indoxyl Sulfate Mediates the Low Inducibility of the NLRP3 Inflammasome in Hemodialysis Patients
Source: Toxins (Basel). 2021 Jan 7;13(1):38. doi: 10.3390/toxins13010038 (PMC7825677; doi:10.3390/toxins13010038)
Supplement: Supplementary file 1 [file toxins-13-00038-s001.pdf]

# Supplementary Materials: Indoxyl Sulfate Mediates the Low Inducibility of the NLRP3 Inflammasome in Hemodialysis Patients

**Li-Chun Ho, Ting-Yun Wu, Tsun-Mei Lin, Hung-Hsiang Liou and Shih-Yuan Hung**

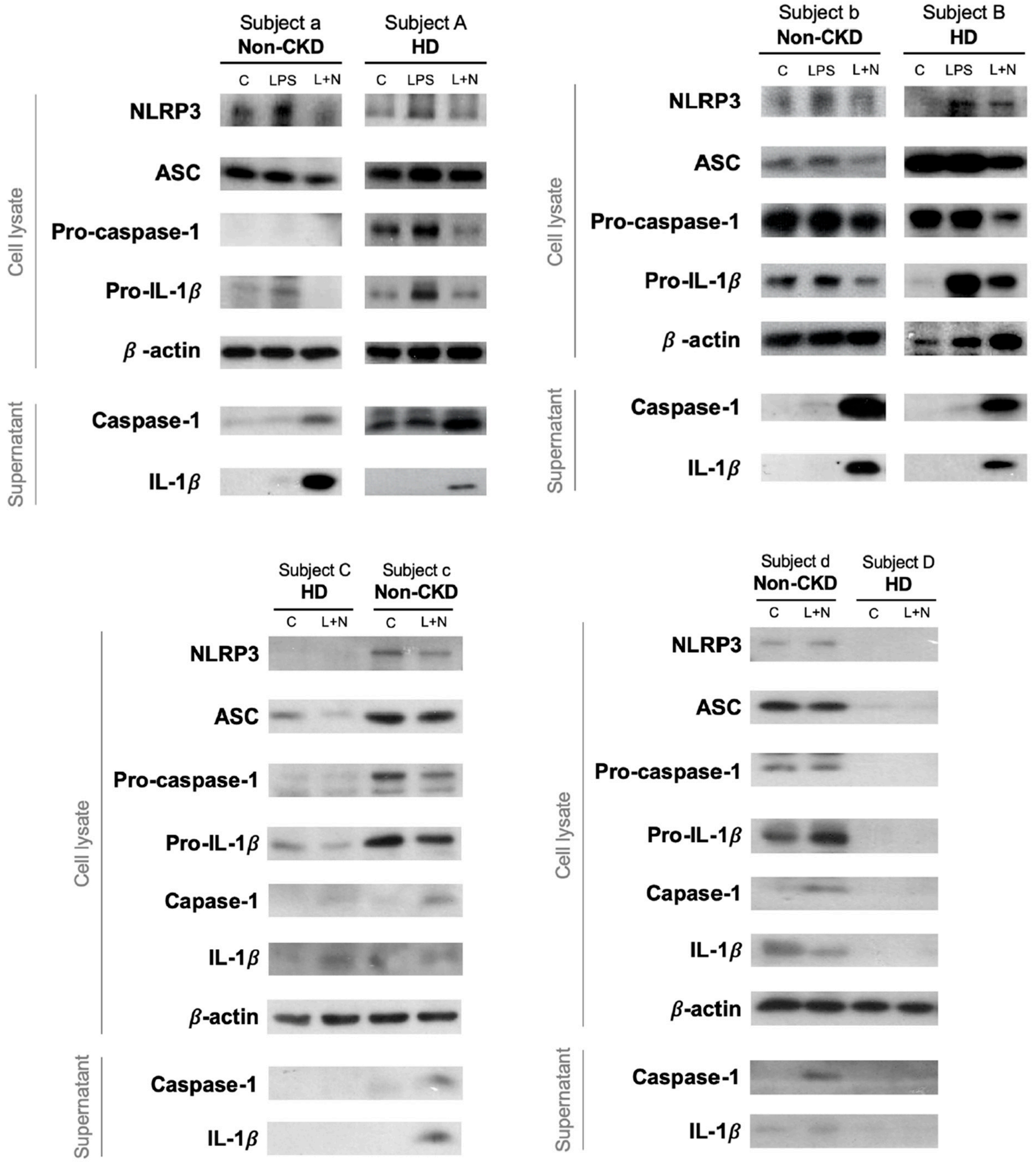


**Figure S1.** PBMC responses to NLRP3 inflammasome induction, 4 illustrated results of immunoblot analysis. PBMCs isolated from each individual were divided either into 3 groups (treated with control medium, LPS for 3.5 hours, or LPS for 3.5 hours plus nigericin for 0.5 hour [subject a, A, b, B]), or into 2 groups (treated

with control medium or LPS plus nigericin [subject C, c, d, D]). The tests for subject a, A, b, and B were performed on different days, while simultaneous PBMC isolation and induction were done on subject C and c and on subject d and D. The latter is to ensure identical experimental conditions for subjects with HD and without CKD. The image of subject C and c is the original images of **Figure 2b**. The images of subject a and A and b and B is the original image of **Figure 3**.

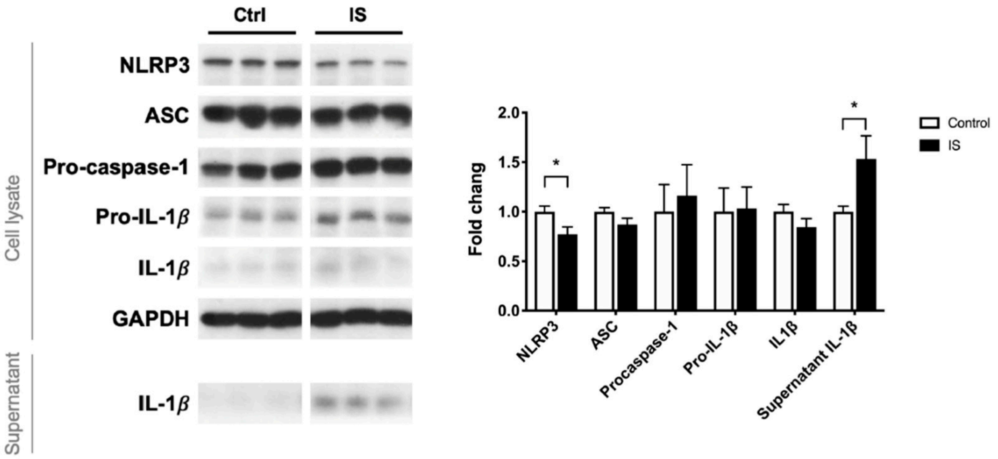


**Figure S2.** Responses of THP-1 derived macrophages to indoxyl sulfate (IS) treatment. The 7 experiments involving the treatment of THP-1-derived macrophages with LPS or LPS plus nigericin all contained the IS treatment group. The figures show the combined results of the IS treatment group, with the representative images and bar graphs showing the quantification of the immunoblot analyses. Control, n = 21; IS, n = 21. *$P \leq 0.05$.

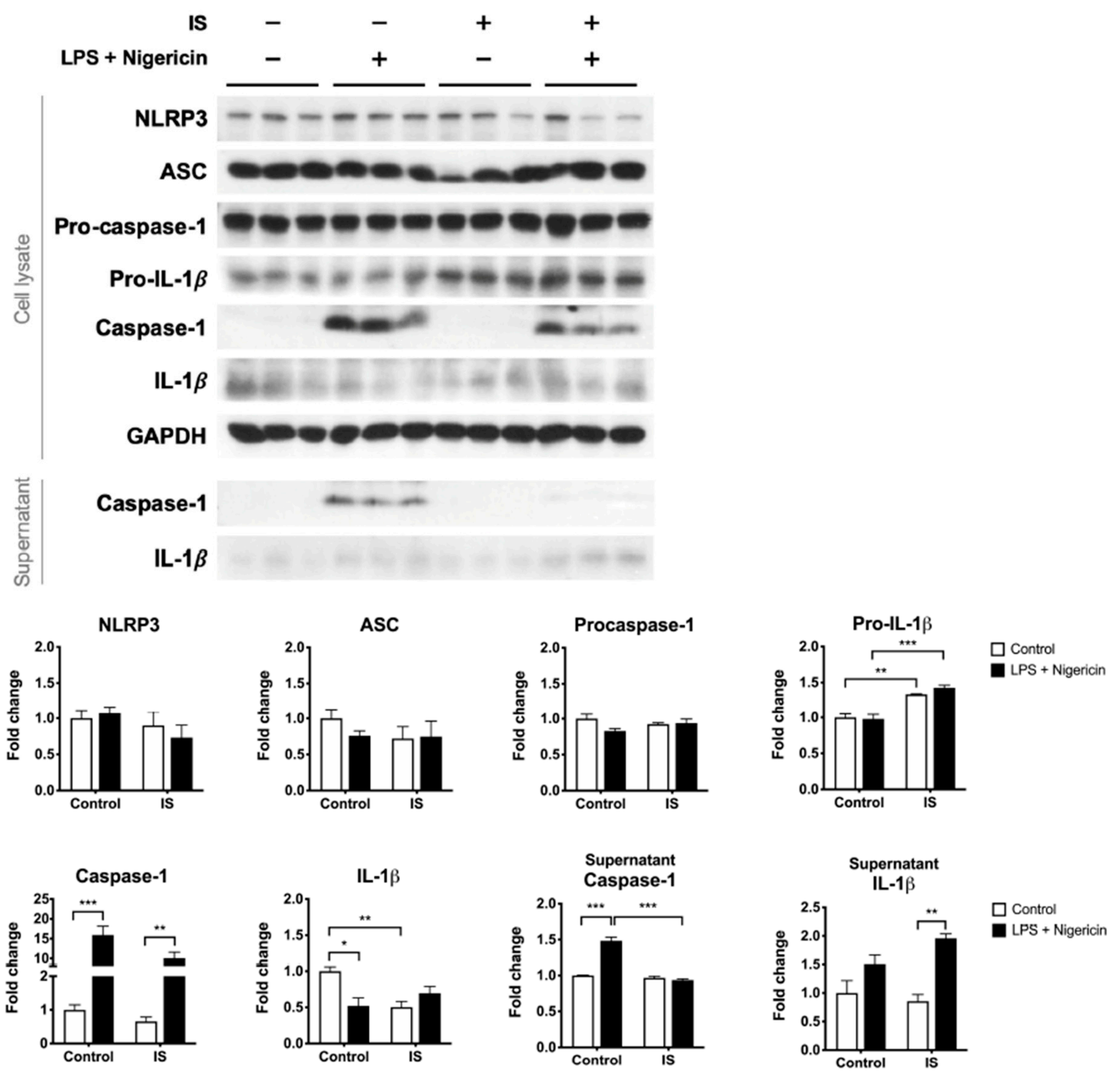


**Figure S3.** Effects of indoxyl sulfate (IS) on the inducibility of NLRP3 inflammasome. THP-1-derived macrophages were treated with control medium or IS for 24 hours and then with 100 ng/mL LPS plus 1 μg/mL nigericin for NLRP3 inflammasome induction; n = 3 for each group. *$P \leq 0.05$, **$P \leq 0.01$, ***$P \leq 0.001$.

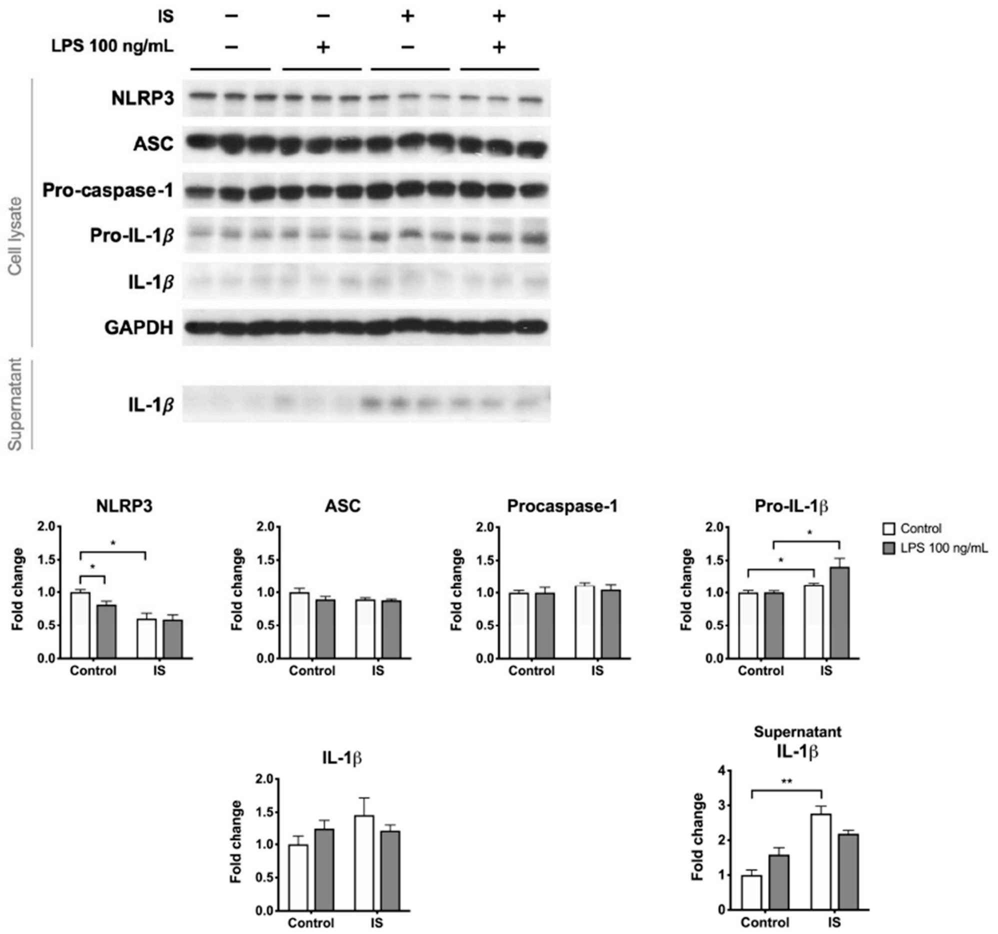


**Figure S4.** Effects of indoxyl sulfate (IS) on the responses to the priming stimulus. THP-1-derived macrophages were treated with control medium or primed with 100 ng/mL LPS with or without pretreatment with IS for 24 hours; n = 3 for each group. **$P \leq 0.01$, ***$P \leq 0.001$.
